# Unconventional transformation of spin Dirac phase across a topological quantum phase transition

**DOI:** 10.1038/ncomms7870

**Published:** 2015-04-17

**Authors:** Su-Yang Xu, Madhab Neupane, Ilya Belopolski, Chang Liu, Nasser Alidoust, Guang Bian, Shuang Jia, Gabriel Landolt, Batosz Slomski, J. Hugo Dil, Pavel P. Shibayev, Susmita Basak, Tay-Rong Chang, Horng-Tay Jeng, Robert J. Cava, Hsin Lin, Arun Bansil, M. Zahid Hasan

**Affiliations:** 1 Department of Physics, Joseph Henry Laboratory, Princeton University, Princeton, New Jersey 08544, USA; 2 Department of Chemistry, Princeton University, Princeton, New Jersey 08544, USA; 3 International Center for Quantum Materials, Peking University, Beijing 100871, China; 4 Swiss Light Source, Paul Scherrer Institute, CH-5232 Villigen, Switzerland; 5 Physik-Institute, Universitat Zurich-Irchel, CH-8057 Zurich, Switzerland; 6 Institute of Condensed Matter Physics, Ecole Polytechnique Fédeérale de Lausanne, CH-1015 Lausanne, Switzerland; 7 Department of Physics, Northeastern University, Boston, Massachusetts 02115, USA; 8 Department of Physics, National Tsing Hua University, Hsinchu 30013, Taiwan; 9 Institute of Physics, Academia Sinica, Taipei 11529, Taiwan; 10 Graphene Research Centre and Department of Physics, National University of Singapore, Singapore 11754, Singapore; 11 Princeton Center for Complex Materials, Princeton Institute for Science and Technology of Materials, Princeton University, Princeton, New Jersey 08544, USA

## Abstract

The topology of a topological material can be encoded in its surface states. These surface states can only be removed by a bulk topological quantum phase transition into a trivial phase. Here we use photoemission spectroscopy to image the formation of protected surface states in a topological insulator as we chemically tune the system through a topological transition. Surprisingly, we discover an exotic spin-momentum locked, gapped surface state in the trivial phase that shares many important properties with the actual topological surface state in anticipation of the change of topology. Using a spin-resolved measurement, we show that apart from a surface bandgap these states develop spin textures similar to the topological surface states well before the transition. Our results offer a general paradigm for understanding how surface states in topological phases arise from a quantum phase transition and are suggestive for the future realization of Weyl arcs, condensed matter supersymmetry and other fascinating phenomena in the vicinity of a quantum criticality.

## Abstract (web summary)

In topological insulators, topology imposes a quantum phase transition between the trivial and nontrivial phases. Here, Xu *et al.* demonstrate how properties of the topological surface states emerge in the trivial phase of BiTl(S_1-δ_Se_δ_)_2_ when close to its chemically tuned phase transition.

## Graphical abstract

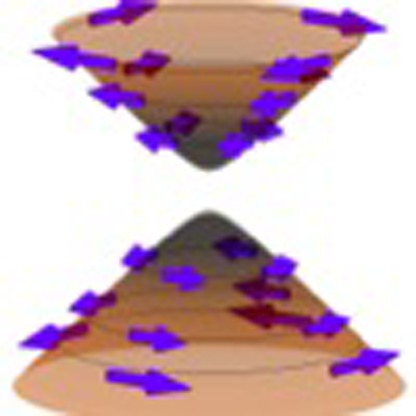

Understanding the physics of distinct phases of matter is one of the most important goals in physics in general. For a new phase of matter, a powerful route towards such an understanding is to study the way in which it arises from an understood state by investigating the nature of a phase transition. A topological insulator (TI), a weakly interacting electronic system, is a distinct phase of matter that cannot be adiabatically connected to a conventional material without going through a topological quantum (*T*→0 K) phase transition (TQPT), which involves a change of the bulk topological invariant without invoking any many body interaction. The discovery of the 3D Z_2_ TI state has attracted huge interest and led to a surge of research in finding new and engineered topological states^1^,^2^,^3^,^4^,^5^,^6^,^7^,^8^,^9^,^10^,^11^,^12^,^13^,^14^,^15^,^16^,^17^,^18^,^19^,^20^,^21^,^22^,^23^,^24^. Many new topological phases of matter, such as a topological crystalline insulator^9^,^10^,^11^, a topological Kondo insulator^12^,^13^,^14^,^15^, a topological Dirac/Weyl semimetal^16^,^17^,^18^,^19^,^20^,^21^,^22^ and so on have just been predicted or realized. All these phases are predicted to feature protected surface states, which serve as the experimental signature for their nontrivial topology in the bulk, and they are in fact formed via TQPTs and need to be understood in real materials. Therefore, it is of general importance to study how protected surface state emerge from a trivial material by crossing the topological critical point (TCP) of a TQPT. However, to date, the electronic and spin groundstate in the vicinity of a TCP for any topological systems remains elusive.

As an example, for a Z_2_ TI, it is well established that the odd number of Dirac surface states and their spin-momentum locking are the signature that distinguishes it from a conventional insulator. However, an interesting and vital question that remains unanswered is how topological surface states emerge as a non-topological system approaches and crosses the TCP. The most straightforward answer is that there are neither surface states nor spin polarization in the conventional insulator (non-topological) regime. In this case, the gapless topological surface states and spin-momentum locking set in abruptly and concomitantly at the TCP. However, there might be more exotic scenarios. Therefore an experimental study focused on this topic is needed to settle this issue.

Understanding the nature of a TCP is also of broad interest because recent theories have proposed a wide range of exciting quantum phenomena based on topological criticality. It has been proposed that the TCP of various TQPTs can not only realize new groundstates such as higher dimensional Dirac fermions^16^,^17^, Weyl fermions under magnetization^16^,^17^,^23^,^24^, supersymmetry SUSY state^4^ and interacting topological states^5^, but also show exotic transport and optical responses such as chiral anomaly in magnetoresistance^25^ or the light-induced Floquet TI state^26^. To achieve them in real materials, it is also quite suggestive to study the electronic and spin groundstate in the vicinity of the TCP in some great detail.

In this article, we report the observation of an exotic phenomenon associated with the formation of the topological surface states across a TQPT. We show that there exists spin-momentum locked but gapped surface states on the topologically trivial side of the TQPT that serves as novel precursor states to the topological surface states. These surface states are systematically enhanced and evolve into the actual topological surface states across the TCP. This is particularly interesting because it can be viewed as a novel proximity effect due to the adjacent TI phase. To achieve these, we systematically study the evolution of electronic and spin groundstate near the TCP with a step finer than 2% in the prototypical TQPT BiTl(S_1−*δ*_Se_*δ*_)_2_ system. The BiTl(S_1−*δ*_Se_*δ*_)_2_ system is known to host one of the most basic TQPTs between a conventional band insulator and a 3D Z_2_ TI^27^,^28^,^29^, and is therefore an ideal platform for our goal. We show that even though the bulk material of BiTl(S_1−*δ*_Se_*δ*_)_2_ lies in the conventional semiconductor regime, we observe an unexpected gapped quasi-two-dimensional (2D) electron gas that shares many properties with actual topological surface states. Surprisingly, our critical spin-resolved (SR) data reveal that these gapped states carry spin polarization, whose momentum space texture at the native Fermi level resembles that of on the surface of a TI. We further show that the observed spin-textured surface states prominently dominate the surface low-energy physics on approaching the TCP, and systematically evolve into the gapless topological surface states. Our observation sets a general paradigm for understanding how topological surface states can arise from a conventional material by going through a TQPT, which is of value for studying various new topological phases and the formation of their protected surface states^9^,^10^,^11^,^12^,^13^,^14^,^15^,^16^,^17^,^18^,^19^,^20^,^21^,^22^,^23^,^24^. The gapped spin-helical surface states also suggest the remarkable potential for the utilization of unique gapped spin-textured electrons on the surface of a carefully designed conventional semiconductor using spin-polarized tunnelling or band-selective optical methods in future applications.

## Results

### Spin-integrated electronic structure near the TCP

**Figure 1:**
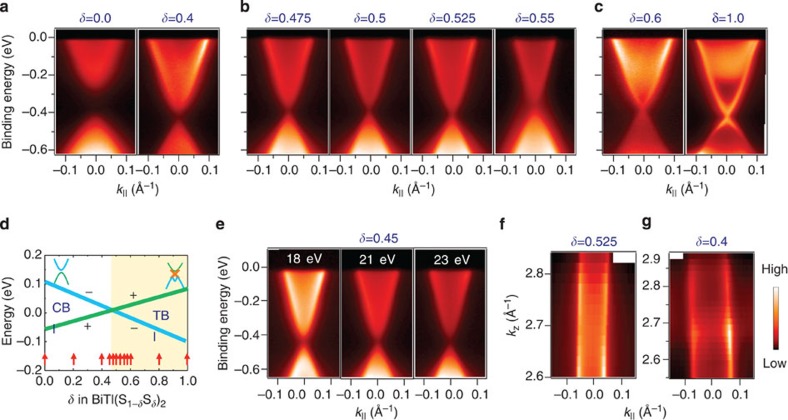
Observation of gapped quasi-2D states before the topological critical point (TCP) of the topological quantum phase transition (TQPT). (**a**–**c**), ARPES *k*_||_-*E*_B_ maps of BiTl(S_1−*δ*_Se_*δ*_)_2_ obtained using incident photon energy of 16 eV. The nominal composition values (defined by the mixture weight ratio between the elements before the growth) are noted on the samples. For a, conventional band insulator, a band gap is clearly observed for *δ*=0.0–0.4; For **b**, compositions near the topological critical point of the topological quantum phase transition, *δ*=0.45, 0.50, 0.525 and 0.55; And for **c**, topological band insulator, the conduction and valence bands are observed to be well separated with the surface states connecting the band gap for *δ*=0.6–1.0. (**d**) The energy levels of the first-principles calculated bulk conduction and valence bands of the two end compounds (*δ*=0.0 and 1.0) are connected by straight lines to denote the evolution of the bulk bands. The compositions selected for detailed experimental studies are marked by red arrows. The + and − signs represent the odd and even parity eigenvalues of the lowest lying conduction and valence bands of BiTl(S_1−*δ*_Se_*δ*_)_2_. (**e**) Incident photon energy-dependence spectra for *δ*=0.45. (**f**,**g**) *k*_*z*_ versus *k*_||_ Fermi surface maps for *δ*=0.525 and 0.4. The *k*_*z*_ range shown for *δ*=0.4 samples corresponds to the incident photon energy from 14 to 26 eV.

We present in-plane electronic structure (*E*_B_ versus *k*_||_) of the BiTl(S_1−*δ*_Se_*δ*_)_2_ system at varying compositions (*δ*). Figure 1 shows that the two end compounds (*δ*=0.0 and 1.0) are in clear contrast, namely, *δ*=0.0 has no surface states and *δ*=1.0 has surface states connecting the bulk conduction and valence bands, which clearly reveals the difference between the conventional semiconductor phase and the Z_2_ topological band insulator phase, in agreement with the previous studies^27^,^28^. The conventional semiconductor state is found to extend from *δ*=0.0 to 0.4 (Fig. 1a), whereas the topological state is clearly observed from *δ*=1.0 to 0.6 (Fig. 1c). A small but observable bulk bandgap of ∼30 meV is observed for *δ*=0.45 in Fig. 1e, indicating that the system continues to belong to the conventional semiconductor phase. Upon increasing *δ* to the region of 0.475–0.525, the bands are found to further approach each other, and the linear dispersion behaviour of the bands is observed to persist at energies all the way across the node (the Dirac point). Thus based on the observed linear dispersion, the critical composition can be estimated to be *δ*_*c*_=0.5±0.03. At *δ*=0.60 (Fig. 1c), a clear bulk conduction band is observed inside the surface states' upper Dirac cone. Moreover, the bulk conduction and valence bands are separated by an observable bulk gap, which is traversed by the gapless topological surface states. Thus, our data show that the system belongs to the TI regime for compositions of *δ* ≥0.60. As for the system lying very close to the bulk inversion at *δ*=0.50 or 0.525, based on the in-plane dispersion data in Fig. 1b alone, the nature of the observed Dirac-like band cannot be conclusively determined, because it can be interpreted as 2D topological surface states or 3D bulk Dirac states^17^ expected near the bulk band inversion. However, one of the two possibilities can be identified by measuring the dispersion along the out-of-plane *k*_*z*_ direction, since the 3D bulk Dirac states are expected to be highly dispersive^18^,^20^,^21^,^27^ (the velocity along *k*_*z*_ direction of the 3D bulk Dirac band at *δ*_*c*_ is estimated to be ∼2.5 eV·Å^−1^ (ref. 27)), whereas the 2D surface states are not expected to show observable dispersion along the *k*_*z*_ direction.

Thus, to better understand the nature of the bands at compositions near the TCP, we perform angle-resolved photoemission spectroscopy (ARPES) measurements as a function of incident photon energy values (Fig. 1e,f,g) to probe their out-of-plane *k*_*z*_ dispersion. On varying the photon energy, one can effectively probe the electronic structure at different out-of-plane momentum *k*_*z*_ values in a 3D Brillouin zone. Fig. 1f shows the incident photon energy (*k*_*z*_) measurements at *δ*=0.525 by the Fermi surface mapping in *k*_||_ versus *k*_*z*_ momentum space. The straight Fermi lines that run parallel to the *k*_*z*_ axis show nearly absence of observable *k*_*z*_ dispersion. Similarly, incident photon energy measurements are also performed at compositions *δ*=0.40 and 0.45, where a clear bulk bandgap is observed (*ν*_0_=0). Surprisingly, even for the gapped electronic structure at *δ*=0.40, 0.45, our data show the clear absence of *k*_*z*_ dispersion. Therefore the observed bands cannot be interpreted as 3D bulk Dirac bands expected near the bulk band inversion. In fact, our systematic *k*_*z*_ measurements (see Supplementary Fig. 1 for more data from 14 to 70 eV) reveal that the electronic states near the Fermi level of the *δ*=0.40,0.45 samples are 2D. Due to the coexistence of bulk states at the same energy, it is most precise to name them as quasi 2D states. But because they are strongly localized near the surface of the sample and because they smoothly evolve into the topological surface states as the system is tuned across the TQPT, we believe that it is also reasonable to call them as surface states. Such anomalously strong surface states on the trivial side suggest that these states are due to their proximity to the TI regime.

### SR measurements below and close to the TCP

**Figure 2:**
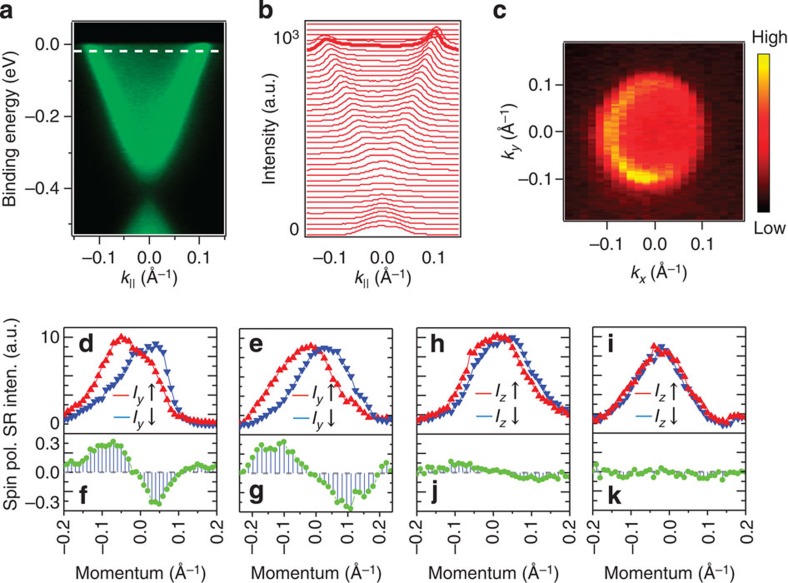
Observation of spin-momentum locking behaviour on the native Fermi level of the gapped surface states in the conventional semiconductor regime. (**a**), ARPES *k*_||_-*E*_B_ map of BiTl(S_1−*δ*_Se_*δ*_)_2_ for a *δ*=0.40 sample. *δ*=0.40 corresponds to a composition in vicinity of the topological phase transition on the trivial side. Dotted line shows the binding energy where the SR measurements (**d**–**k**,) are performed. (**b**) Momentum distribution curves (MDCs) of the dispersion map in**a**. Highlighted MDC is chosen for SR measurements. (**c**) Fermi surface mapping for *δ*=0.40. The two SR measurements are along the 
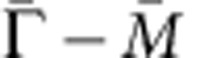
 and 
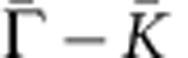
 cuts, respectively. Yellow arrows represent the measured spin polarization (spin pol.) vectors around the Fermi surface. (**d**,**e**) measured in-plane SR momentum distribution spectra along the 
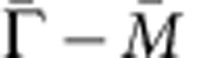
 (**d**) and 
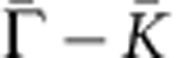
 (**e**) cuts. (**f**,**g**) measured the in-plane net spin polarization along the along the 
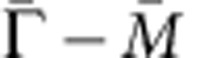
 (**f**) and 
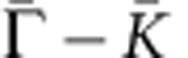
 (**g**) cuts. (**h**–**k**) Same as **d**–**g** but for the out-of-plane component of the spin polarization. *I*_*y*_ ↑ denote the photoemission intensity whose spin polarization is along the positive direction (↑) of the in-plane tangential (*y*) axes.

To study the spin properties of the observed anomalous surface states, we perform SR measurements on the system with compositions below and near the TCP. We present SR data taken on the composition of *δ*=0.40 (Fig. 2a) and focus on the vicinity of the Fermi level (*E*_B_=−0.02 eV). The momentum distribution curves for the spectrum are shown in Fig. 2b, where the highlighted curve is chosen for SR measurements. Figure 2d–g shows the in-plane SR-momentum distribution curve spectra as well as the measured in-plane spin polarization along the 
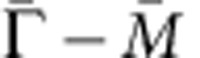
 and 
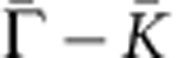
 momentum space cuts. Clear in-plane spin polarization is observed on the surface states from Fig. 2d–g. Furthermore, the measured spin polarization in Fig. 2d–g shows that the spin texture is arranged in a way that spins have opposite directions on the opposite sides of the Fermi surface. In addition, the out-of-plane component of the spin polarization along the 
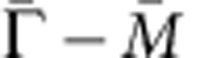
 and 
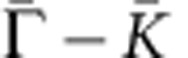
 cuts is shown in Fig. 2h–k. No significant out-of-plane spin polarization (Fig. 2h–k) is observed within our experimental resolution. The spin texture configuration can be obtained from the SR measurements in Fig. 2d–k, as schematically shown by the arrows in Fig. 2c. Surprisingly, our SR measurements reveal that these surface states are not only strongly spin polarized, but their spin texture near the native Fermi level resembles the helical spin texture on the topological surface states as observed in Bi_2_Se_3_ (ref. 8).

**Figure 3:**
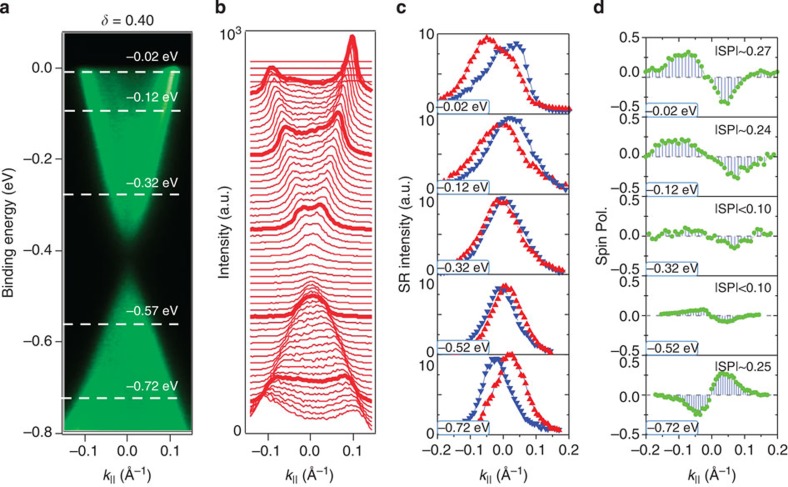
Evolution of the surface states' spin polarization with binding energy. (**a**) ARPES *k*_||_-*E*_B_ map of the *δ*=0.40 sample with dotted lines indicating the energy levels of SR measurements. (**b**) Momentum distribution curves with highlighted curves chosen for SR measurements. (**c**) SR momentum distribution spectra and (**d**) the corresponding net spin polarization measurements.

**Figure 4:**
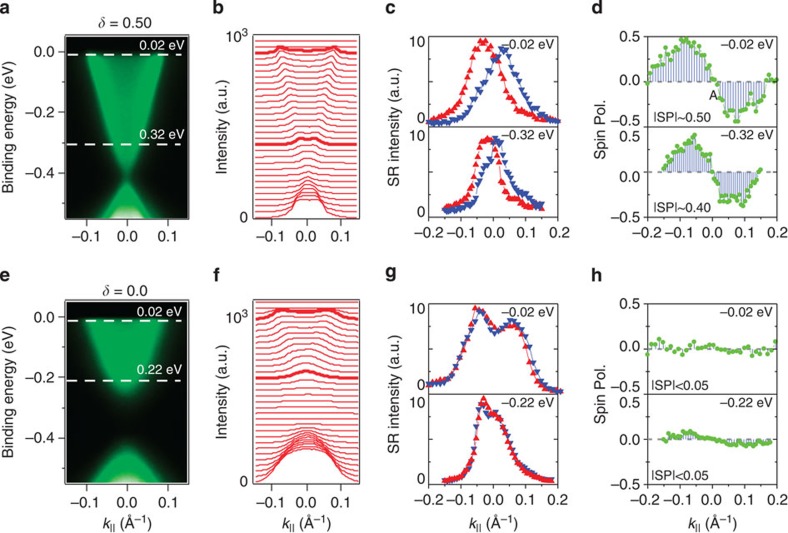
Evolution of the surface states' spin polarization with composition. (**a**,**e**) ARPES *k*_||_-*E*_B_ maps with dotted lines indicating the energy levels of SR measurements. Compositions of the samples are marked on the top of each map. (**b**,**f**) Momentum distribution curves with highlighted curves chosen for SR measurements. (**c**,**g**) SR momentum distribution spectra and (**d**,**h**) the corresponding net spin polarization measurements.

We present systematic SR studies to understand the way spin texture of the surface states evolves as a function of binding energy *E*_B_ and composition *δ*. Figure 3a–d shows SR data at different binding energies for a sample with *δ*=0.40. The spin-momentum locking behaviour is observed at all binding energies from near the Fermi level (*E*_B_=−0.02 eV) to an energy near the conduction band minimum (*E*_B_=−0.32 eV). While the magnitude of the spin polarization on the Fermi level is found to be around 0.3, the spin polarization magnitude is found to decrease to nearly zero while approaching small values of momenta near the Kramers' point 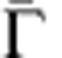 (the conduction band minimum). Furthermore, at energies cutting across the bulk valence band at *E*_B_=−0.57 eV, *E*_B_=−0.72 eV, the measured spin polarization profile is clearly reversed, where a right-handed profile is found for the surface states on the boundary. In addition, the magnitude of the spin polarization is found to be increased as the energy is tuned away from the bulk bandgap, which is consistent with the gapped nature of the surface states. The observed reduction of net spin polarization at a small momenta and the absence of net spin polarization at the 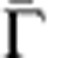 (*k*=0, see Supplementary Fig. 2) point are important for the gapped nature of surface states in *δ*=0.4 samples. As for the gapless case with the system composition at *δ*=0.50, the SR measurements (Fig. 4a–d) reveal the same helical-like spin texture configuration on the Fermi level, where the magnitude of the spin polarization is around 0.5 at the Fermi level in this composition. However, in contrast to the *δ*=0.4 case, it does not show any obvious reduction in going to small values of momenta near the Kramers' point 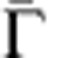 (spin polarization ∼0.45 for *E*_B_=−0.32 eV), which is consistent with its gapless nature. The adequate energy-momentum resolution of our SR-ARPES instrument, to resolve opposite spins at small momenta, such as *k*∼0.05 Å^−1^, is demonstrated by these SR measurements on *δ*=0.50, which strongly supports that the observed strong spin polarization reduction at the *δ*=0.40 case reveals an intrinsic property of the system relevant to the topological transition. Finally, we present the spin data taken on the composition far into the topologically trivial side (*δ*=0.0). Our SR measurements (Fig. 4e–h) show only very weak polarizations (∼0.05), which lie within the uncertainty levels of the measurements. The magnitude of the spin polarization is too weak (comparable to the instrumental resolution) to obtain the spin texture configuration around the Fermi surface for samples with *δ*=0.0. The observed weak polarization on *δ*=0.0 suggests that the surface states are much suppressed in going away from the TCP (such as the *δ*=0.0 samples). More systematic SR studies can be found in Supplementary Figs 2,3.

In Supplementary Discussion and Methods, we model the surface of a topological phase transition system based on the 4 × 4 *k*·*p* model (ref. 30) and utilize the Green's function method to obtain the spectral weight as well as the spin polarization near the surface region of the system as a function of bulk bandgap value in the model. We found a reasonable qualitative agreement between our experimental results and the *k*·*p* model calculation as seen in Supplementary Fig. 4.

## Discussion

Although the observed surface states share important properties with actual topological surface states, the following observations from our data clearly show that they are still consistent with the non-topological bulk regime. First, the experimentally observed surface states are gapped and disperse roughly along the edge of the bulk continuum. Thus they do not connect or thread states across the bulk bandgap as in a Z_2_ TI. Second, it is also possible to choose an energy value within the bulk bandgap for samples lying in the conventional semiconductor regime (for example, *δ*=0.4), so that no surface state is traversed, consistent with the topological triviality of the sample. These experimental facts guarantee that the observed surface states at 
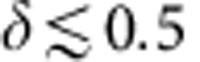
 are consistent with the conventional semiconductor phase of the system (*ν*_0_=0, trivial Z_2_ index). In the Supplementary Discussion and Supplementary Figs 5,6, we propose a phenomenological picture that involves a Rashba-like state for our observed spin-momentum locked surface states on the trivial side before the TQPT. Nevertheless, the detailed theoretical understanding requires a microscopic theory to show why spin-momentum locking is formed even before the TQPT actually takes place. Regardless of the theoretical studies required in the future, our observations experimentally reveal a novel proximity effect due to the adjacent TI phase in a TQPT system. First, the surface states on the trivial side show spin polarization texture that resembles a TI. Second, their surface spectral weight and magnitude of spin polarization are enhanced as we approach the TCP. Third, they evolve into the topological surface states. These measurements with spin and momentum resolution clearly show that these surface states are critically relevant to the bulk band inversion and TQPT in the bulk. In the Supplementary Fig. 7, we show similar observation near the TCP of another prototypical TQPT system (Bi_1−*δ*_In_*δ*_)_2_Se_3_ (ref. 29). Therefore, these systematic and careful measurements on multiple systems suggest that our observation is unlikely a special case due to material details of the BiTl(S_1−*δ*_Se_*δ*_)_2_ system but an important proximity phenomenon that describes the TCP in the electronic and spin groundstates in many TQPT systems. Our observation can also be applied to explain a number of recent experiments on some newly predicted topological matter, such as the topological Kondo insulator phase predicted in SmB_6_ (ref. 12). In SmB_6_, the Kondo hybridization gap is believed to become significant below 30 K and the low-temperature resistivity anomaly occurs below 6 K (ref. 13). However, ARPES experiments have observed quasi 2D low-energy states without *k*_*z*_ dispersion persisting up to temperatures ≥100 K (refs 14, 15). Furthermore, a recent theoretical effort proposed that the formation of spin-momentum locked surface states before the TQPT is due to the reversal of bulk Dirac fermion chirality across the TQPT^31^ with consequences for optics^32^, which is consistent with our systematic experimental data. We also note that the quantum fluctuation can be another interesting direction, which is widely studied in many non-topological quantum phase transitions, where a local order parameter is present. However, to study that in our system, it is necessary to first theoretically understand the role of quantum fluctuations in a topological quantum phase transition in a non-interacting or weakly-interacting system. Only then, it is possible to identify the correct experimental probe that can be sensitive to the quantum fluctuation in our studied system.

**Figure 5:**
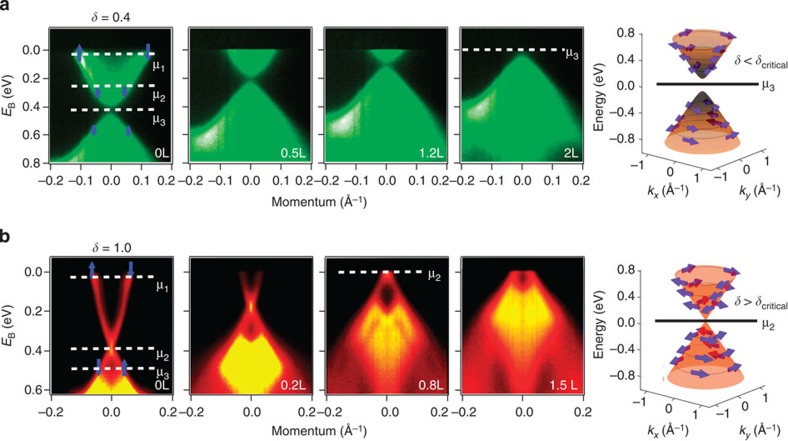
Spin-momentum locked gapped surface states before the TCP realize a helical spin switch in a non-topological and non-magnetic setting. ARPES dispersion measured with incident photon energy of 55 eV under NO_2_ surface adsorption on the *δ*=0.4 (**a**) and *δ*=1.0 (**b**) samples. The NO_2_ dosage is noted on top of each panel. 1 l=1 × 10^−6^ torr s^−1^. Blue arrows represent the measured spin polarization of the sample. The length of the arrow qualitatively show the magnitude of the spin polarization. At the chemical potential of μ_3_ for the *δ*=0.4 sample, a surface insulator is realized. Thus the helical spin texture can be switched on and off by tuning the chemical potential.

Irrespective of the theoretical origin, our observation itself is important for further considerations of these novel states^33^. We propose potential device applications for the spin-textured gapped surface states that we observed. Since there is a true energy gap without any states (neither surface nor bulk) in the conventional insulator phase (such as the *δ*=0.4 sample), thus in this case the spin-textured surface electrons can be turned on and off via tuning the chemical potential of the samples, which realizes a novel switch of the spin-textured surface electrons not possible for a usual topological surface state without adding magnetism. Such a spin switch is experimentally demonstrated in BiTl(S_1−*δ*_Se_*δ*_)_2_ through NO_2_ surface adsorption on a *δ*=0.4 sample under ultra-high vacuum conditions as shown in Fig. 5. Due to the fact that there are bulk bands at the same energy where these surface states exist, they need to be investigated and utilized using surface-sensitive approaches. For example, in tunnelling measurements, they open up new possibilities for observing anomalous behaviour, anomalous transmission near step edges and other unusual surface effects on a conventional semiconductor surface, which can be switched on and off via changing the sample bias in tunnelling experiments. Similarly, our observations also enable band-selective optical experiments, such as photocurrent and photoconductivity manipulation using circularly polarized incident light^32^, leading to potential optospintronics applications. Further research towards these goals will require higher quality nanostructured molecular beam epitaxy grown samples.

## Methods

### Electronic structure measurements

Spin-integrated ARPES measurements were performed with incident photon energy of 8–30 eV at beamline 5-4 at the Stanford Synchrotron Radiation Lightsource (SSRL) in the Stanford Linear Accelerator Center, with 26–90 eV at beamlines 4.0.3, 10.0.1 and 12.0.1 at the Advance Light Source in the Lawrence Berkeley National Laboratory and with 16–50 eV at the PGM beamline in the Synchrotron Radiation Center in Wisconsin. Samples were cleaved *in situ* between 10 and 20 K at a chamber pressure better than 5 × 10^−11^ torr at all endstations at the SSRL, the Advance Light Source and the Synchrotron Radiation Center, resulting in shiny surfaces. Energy resolution was better than 15 meV and momentum resolution was better than 1% of the surface Brillouin zone. Adsorption of NO_2_ molecules on the sample surface was achieved via controlled exposures to NO_2_ gas (Matheson, 99.5%). The adsorption effects were studied under static flow mode by exposing the clean sample surface to the gas for a certain time at the pressure of 1 × 10^−8^ torr, then taking data after the chamber was pumped down to the base pressure. Spectra of the NO_2_ adsorbed surfaces were taken within minutes of opening the photon shutter to minimize the potential photon-induced charge transfer and desorption effects.

### SR measurements

SR ARPES measurements were performed on the SIS beamline at the Swiss Light Source using the COPHEE spectrometer with two 40 kV classical Mott detectors and photon energy of 20–70 eV, which systematically measures all the three components of the spin of the electron (P_*x*_, P_*y*_ and P_*z*_) as a function of its energy and momentum^34^. Energy resolution was better than 60 meV and momentum resolution was better than 3% of the surface Brillouin zone. Samples were cleaved *in situ* at 20 K at chamber pressure <2 × 10^−10^ torr. Typical electron counts on the Mott detector reached 5 × 10^5^, which placed an error bar of ±0.01 for the data points in all the spin polarization measurements. Our SR ARPES measurements were performed with linearly *p*-polarized light at synchrotron radiation energies 20–70 eV, where the final state effects are demonstrated to be negligible^35^.

### Sample growth

Single crystals of BiTl(S_1−*δ*_Se_*δ*_)_2_ were grown from high purity elements mixed in a stoichiometric ratio using the Bridgman method systematically described inrefs 27, 36. The mixture was heated in a clean evacuated quartz tube to 900 °C where it was held for two days. Afterwards, it was cooled slowly, at a rate of 1.5 °C per hour in the vicinity of the melting point, from the high temperature zone towards room temperature. The spatial compositional homogeneity of the cleaved sample surfaces were confirmed using high-resolution energy dispersive spectroscopy (EDS). The EDS measurements were performed at the Imaging and Analysis Center at the Princeton University's Institute for the Science and Technology of Materials. The equipment used was a FEI company Quanta 200 f field emission Environmental Scanning Electron Microscope equipped with an Oxford INCA EDS data analysis software system and an Oxford XMAX 80 mm^2^ high-efficiency EDS detector (see Supplementary Fig. 8,9 and Supplementary Methods for details). We note that the *n*-doping in the samples were caused by Se or S vacancies^8^. And depending on the number of vacancies in a crystal, its chemical potential slightly varied from batch to batch. We did notice a small variation of the chemical potential (for example, see Fig. 1b of the main text). However, since the samples were always *n*-type, we could always observe the conduction band, the valence band, the bandgap, and the surface states in ARPES. Thus the small variation of the chemical potential did not affect our results and conclusions.

### First-principles and model theoretical calculation methods

The theoretical band calculations were performed with the linearized augmented planewave method using the WIEN2K package^37^ within the framework of density functional theory. The generalized gradient approximation was used to model the exchange-correlation effects. For the semi-infinite surface system, model calculations were done based on a Green's function with implementation of the experimentally based *k* · *p* model (ref. 30) to reveal the electronic structure and spin configuration near the surface region of the BiTl(S_1−*δ*_Se_*δ*_)_2_ system.

## Author contributions

S.-Y.X. and M.N. performed the experiments with assistance from I.B., C.L., N.A., G.B., P.P.V. and M.Z.H.; S.J. and R.J.C. provided the samples; G.L., B.S. and J.H.D. assisted with the SR ARPES measurements and the data analysis and interpretation; S.B., T.-R.C., H.-T.J., H.L. and A.B. carried out the theoretical calculations; M.Z.H. was responsible for the overall direction, planning and integration among different research units.

## Additional information

**How to cite this article:** Xu, S.-Y. *et al.* Unconventional transformation of spin Dirac phase across a topological quantum phase transition. *Nat. Commun.* 6:6870 doi: 10.1038/ncomms7870 (2015).

## Supplementary Material

Supplementary Information Supplementary Figures 1-9, Supplementary Discussion, Supplementary Methods and Supplementary References
