# Supplementary material for: Unconventional transformation of spin Dirac phase across a topological quantum phase transition
Source: Nat Commun. 2015 Apr 17;6:6870. doi: 10.1038/ncomms7870 (PMC4410671; doi:10.1038/ncomms7870)
Supplement: Supplementary Information — Supplementary Figures 1-9, Supplementary Discussion, Supplementary Methods and Supplementary References [file ncomms7870-s1.pdf]

Supplementary Figures

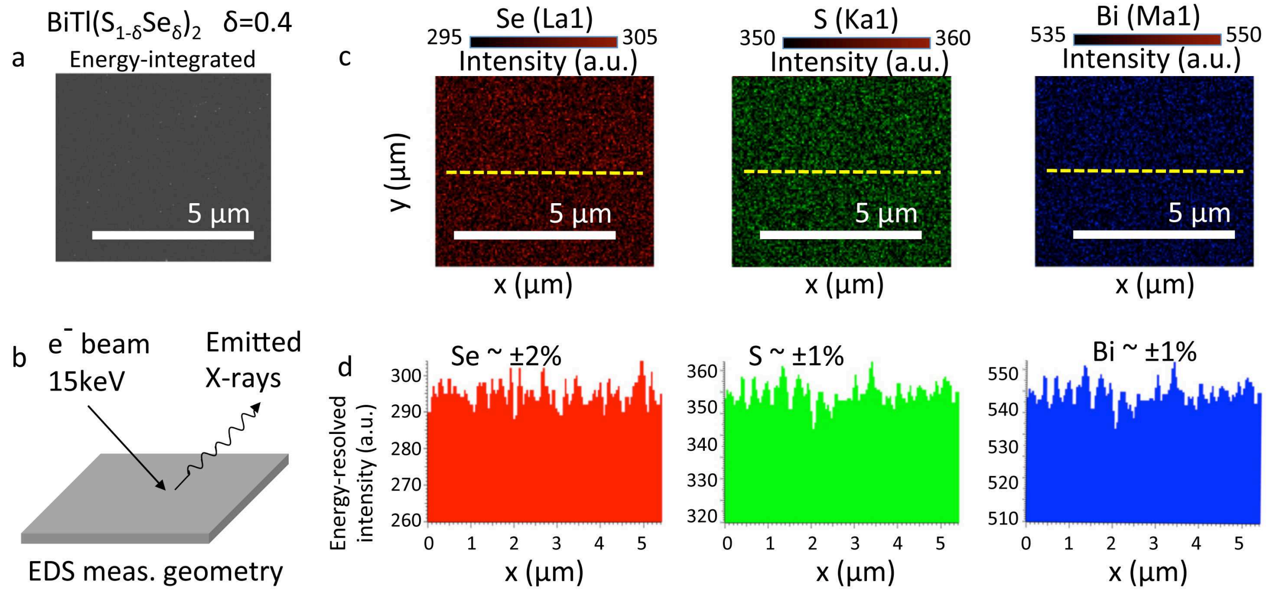

Supplementary Figure 1: **Energy dispersive spectroscopy measurements as a function of real-space location on the cleaved surface of a $\delta = 0.4$ sample. a,** Energy-integrated EDS spectra. The white solid line indicates the scale bar of real space dimension. **b,** Measurement geometry of our EDS experiments. A high-energy electron beam (15keV) is applied to the sample. And the emitted x-ray, characterizing unique core-shell energy levels of the elements in a sample, is detected by an EDS crystal detector. **c,** EDS spectra in x,y real-space at energy levels corresponding to the selenium La1 (M5 to L3) 1379.1 transition, sulfur Ka1 (L3 to K1) 2307.8 eV transition, and bismuth Ma1 (N7 to M5) 2422.6 eV transition. Nearly uniform spectra are revealed for all three different elements. The yellow dotted lines indicate a cut along the x-axis. **d,** EDS spectra along x-axis. The cut is defined by the yellow dotted line in panel **c**.

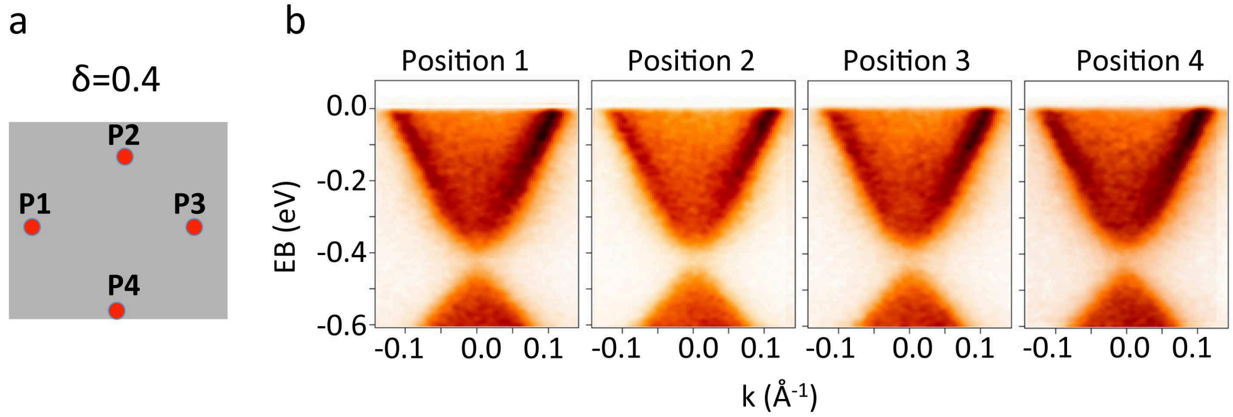

Supplementary Figure 2: ARPES measurements on different locations of a cleaved sample surface. a, Schematic drawing of a cleaved surface of a $\delta = 0.4$ sample. The size of the sample is about 2mm$\times$2mm in x and y dimensions, whereas the ARPES beamspot size is around $200\mu m^2$ to $500\mu m^2$. The four positions noted as P1-P4 are selected for ARPES measurements. **b,** ARPES measured band structure at the four positions.

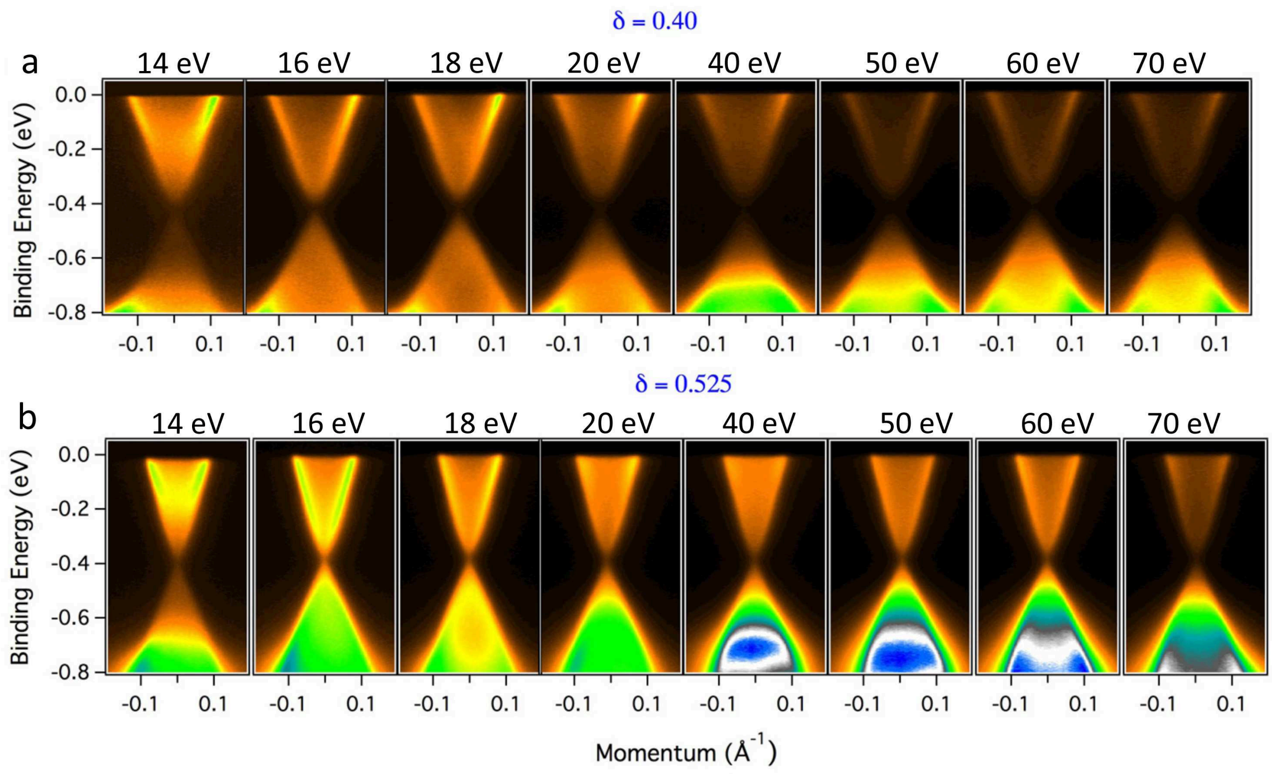

Supplementary Figure 3: **Incident photon energy dependence ARPES spectra. a,** $\delta = 0.40$. **b,** $\delta = 0.525$. The incident photon energy values used for the measurements are noted on the top of each panel.

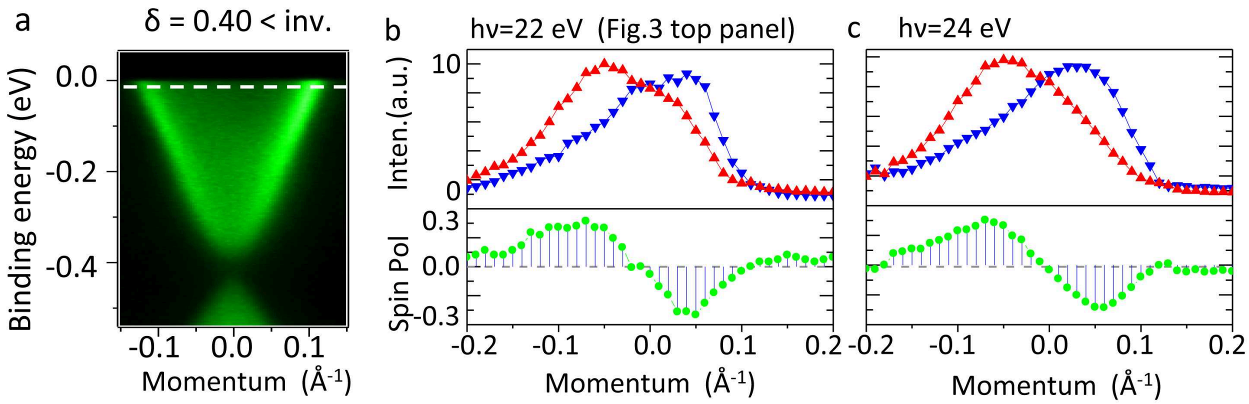

Supplementary Figure 4: **Spin measurements at different photon energy.** Spin-resolved measurements are performed along the $\bar{M}-\bar{\Gamma}-\bar{M}$ direction, on a $\delta = 0.4$ sample at two independent incident photon energies, in order to rule out potential systematic errors in the spin measurements. **a,** Spin-integrated ARPES dispersion map of a $\delta = 0.4$ sample. The white dotted line indicates the binding energy of the SR-measurements shown on the right-hand-side. **b,** SR-measurement using incident photon energy of 22 eV. **c,** SR-measurement under identical experimental conditions but using incident photon energy of 24 eV.

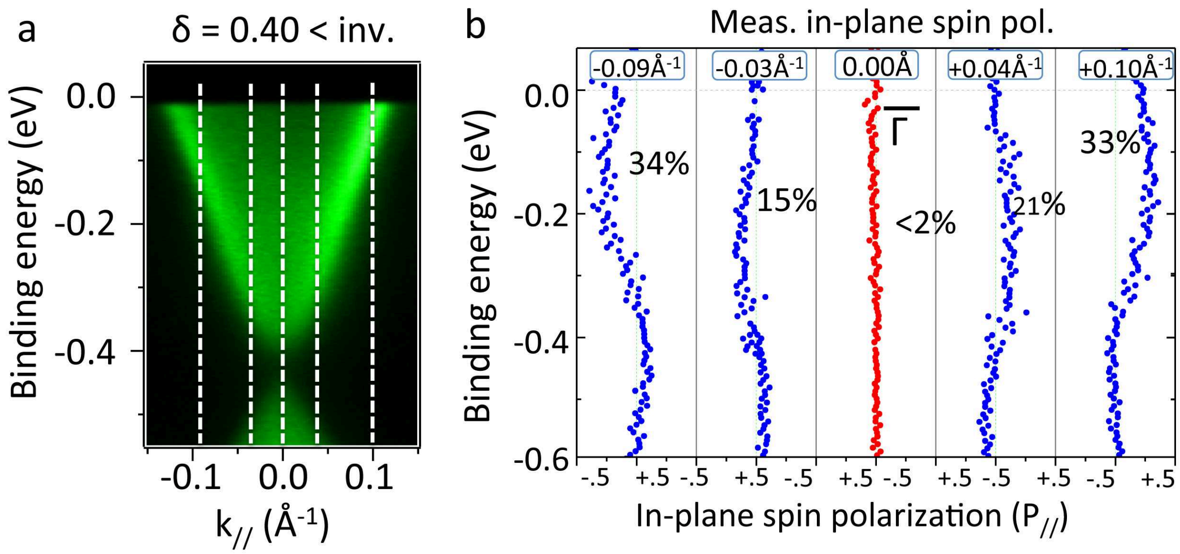

Supplementary Figure 5: **Spin-resolved Measurements with spin-resolved energy distribution curve (SR-EDC) mode on a** $\delta = 0.4$ **sample. a,** Spin-integrated ARPES dispersion map of a $\delta = 0.4$ sample. The white dotted lines indicate the momentum values used for SR-EDC measurements shown in panel **b**. **b,** Measured spin polarization as a function of binding energy at fixed momentum values (noted on top). No observable spin polarization is observed at $k = 0$, demonstrating spin degeneracy at the time-reversal invariant $\bar{\Gamma}$ point.

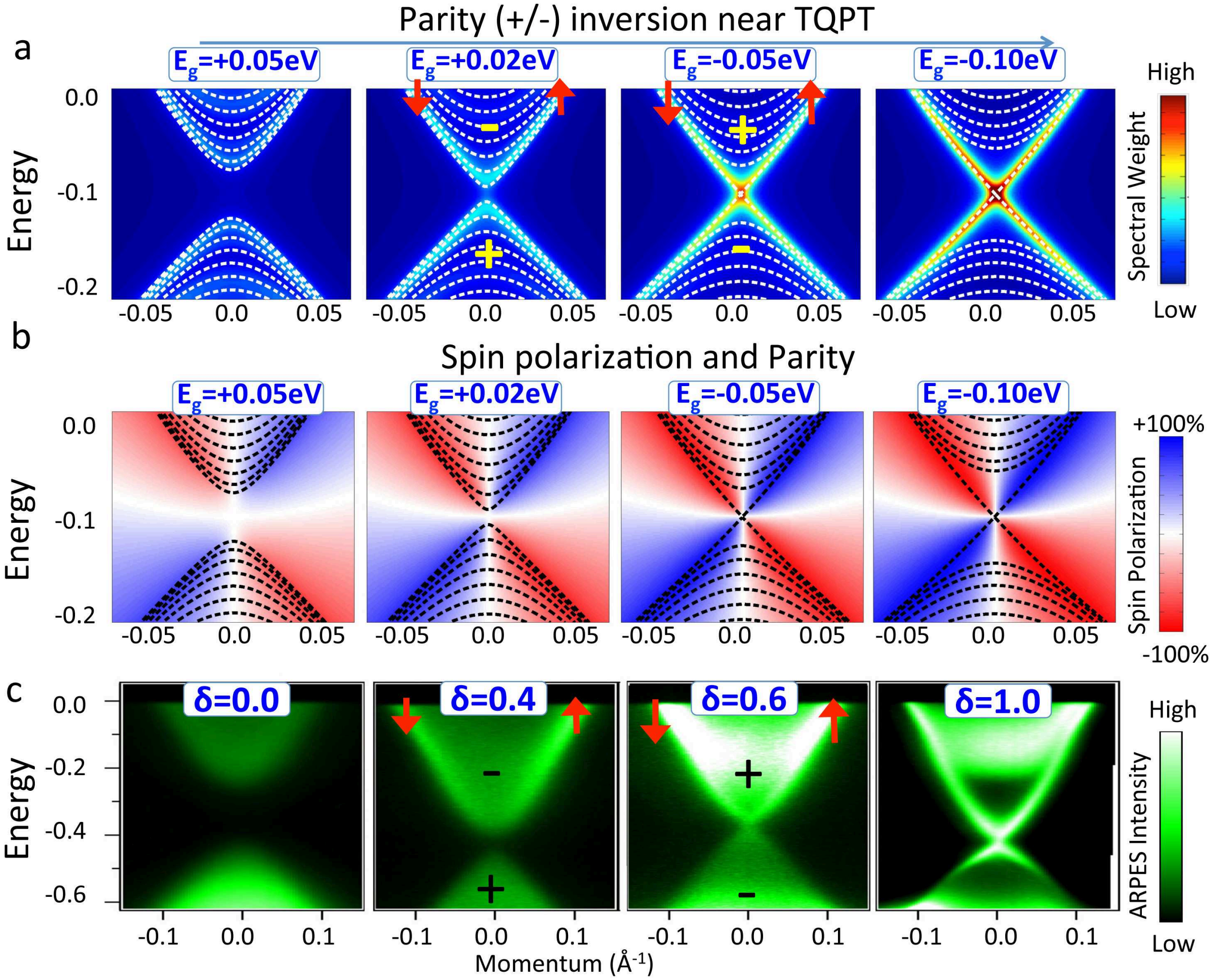

Supplementary Figure 6: Near-surface band structure and spin polarization of BiTl($S_{1-\delta}Se_\delta$)$_2$. a,b, Calculated electronic spectral weight distribution (**a**) and spin polarization (**b**) near the surface region of the constructed system are shown by the color maps in panels (**a** and **b**), respectively. The dispersion of the bulk bands are shown by the white (black) dotted lines. Positive band-gap means that the system lies in the conventional semiconductor (insulator) $\nu_0 = 0$ regime, whereas negative band-gap value means that the system lies in the topological insulator $\nu_0 = 1$ regime. **c,** ARPES measured electronic dispersion on various δ values across the TQPT. The + and − signs in panels (**a** and **c**) represent the odd and even parity eigenvalues of the lowest lying conduction and valence bands of BiTl($S_{1-\delta}Se_\delta$)$_2$. The red ↑ and ↓ arrows schematically show the in-plane spin polarization near the sample surface before and after the bulk band inversion (the TCP).

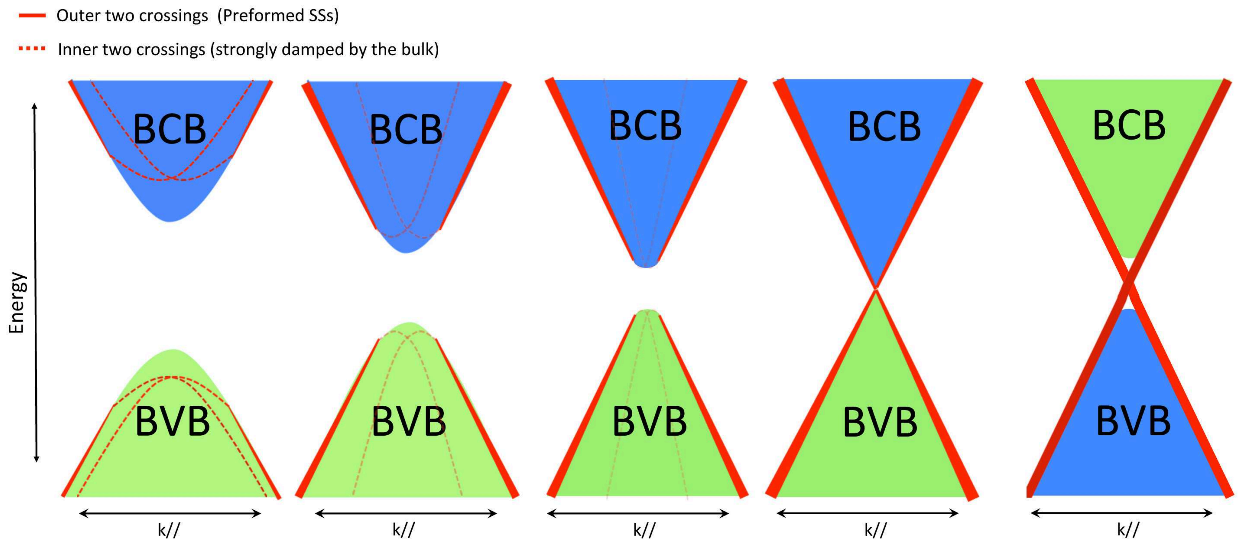

Supplementary Figure 7: A band-like schematic showing the phenomenological picture we proposed in the main text. The blue and green shaded areas represent the bulk conduction bands (BCB) and the bulk valence bands (BVB), which go through a band inversion at the critical point. The red lines represent electronic states that are strongly localized on the surface due to their minimal overlap with the bulk band continuum.

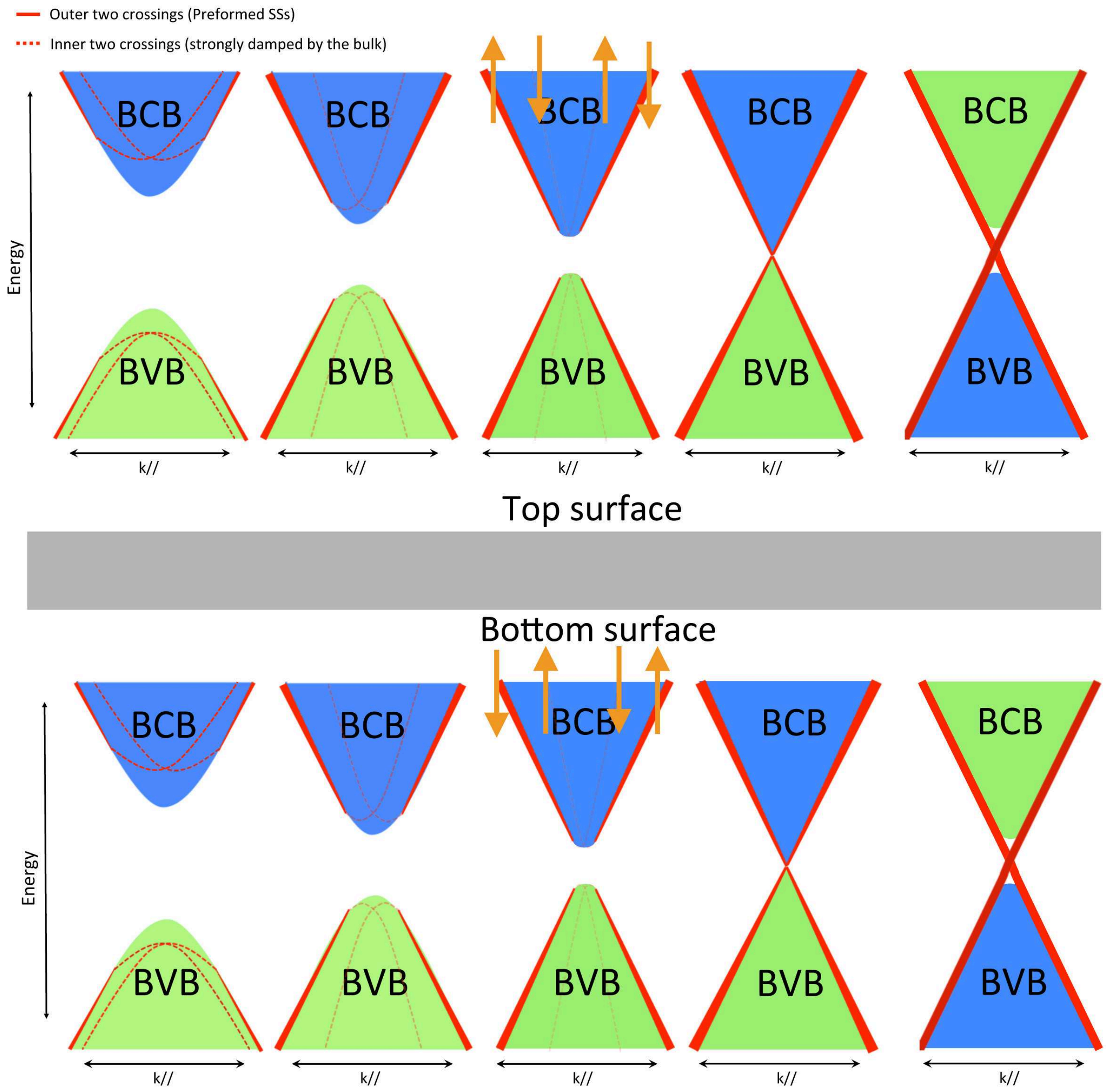

Supplementary Figure 8: The inner band (dotted lines) become bulk-like. One might worry about their singly degenerate nature, which is inconsistent with the doubly degenerate nature of the bulk bands. However, it is important to consider that there is another copy of these bands that has the opposite spin texture one the opposite surface of the sample.

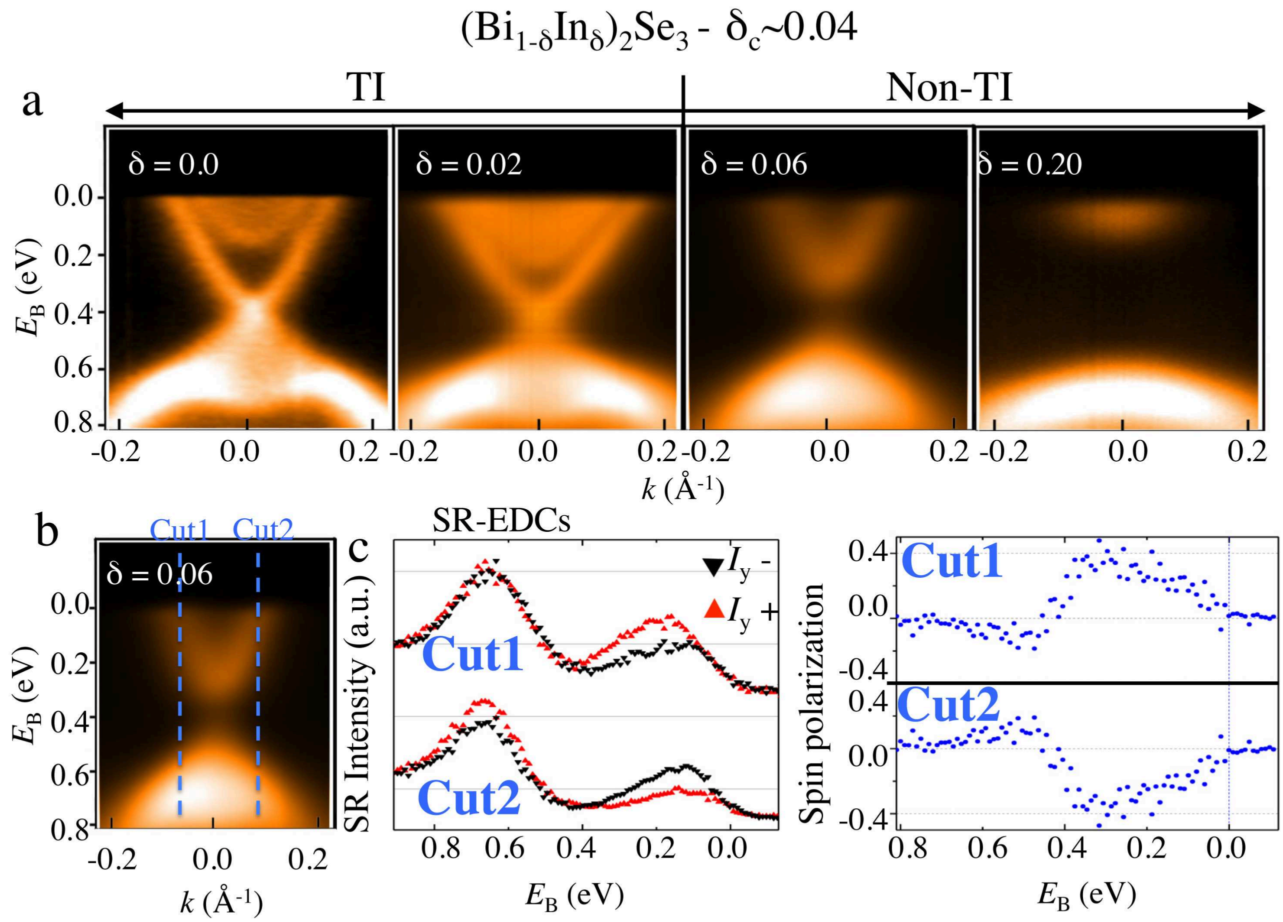

Supplementary Figure 9: **Preformed spin-textured surface states in another topological phase transition system** $(\mathbf{Bi}_{1-\delta}\mathbf{In}_{\delta})_2\mathbf{Se}_3$**. a,** ARPES dispersion maps at various In composition δ values. The critical point of the topological phase transition is about $\delta_c \sim 0.04$. **b,** Dispersion of the composition $\delta = 0.06$ sample repeated. The $\delta = 0.06$ sample is found to be in the conventional semiconductor (topologically trivial) phase. The blue dotted lines define the momenta chosen for spin-resolved ARPES measurements. **c,** Spin-resolved ARPES intensity and net spin polarization for the two spin-resolved cuts.

Supplementary Discussion

- **Spatial homogeneity of the samples**

  Energy dispersive spectroscopic (EDS) measurements were performed on our $BiTl(S_{1-\delta}Se_\delta)_2$ samples in order to check the spatial chemical homogeneity of the samples.

  Supplementary Figure 1 shows the EDS measurements on the cleaved surface of a representative piece of our $\delta = 0.4$ samples used in ARPES experiments. With high-energy (15 keV) electron incident beam, we choose the core level transitions including selenium La1 (M_5 to L_3) transition, sulfur Ka1 (L_3 to K_1) transition, and bismuth Ma1 (N_7 to M_5) transition. Spatially uniform EDS spectra in an energy-resolved (elemental-resolved) fashion are observed across the approximately $5\mu m \times 5\mu m$ measurement window as shown in Supplementary Figure 1c. The energy-resolved EDS intensity fluctuation, which is proportional to the variation of the elemental composition as a function of real-space location, is found to be within $\pm 2\%$. We note that the typical size of the ARPES beamspot ranges from 200 to 500 μm^2, whereas the EDS measurements have a spatial resolution of about 100 nm^2. Thus these measurements rule out the possibility that our results are artifacts due to spatial imhomogeneity of the samples. We note that the n-doping in the samples are caused by Se vacancies. And depending on the number of vacancies in a crystal, its chemical potential will slightly vary from batch to batch. We do notice a small variation of the chemical potential (Fig. 1b of the main text).

  In addition to the EDS measurements, we have also performed ARPES measurements on different locations of the same cleaved surface of each sample studied, in order to check the *in-situ* spatial homogeneity in our ARPES experiments. Supplementary Figure 2 shows the ARPES measured dispersion at 4 different locations on the cleaved surface of a $\delta = 0.4$ sample. By moving the ARPES beamspot around the sample surface, consistent results without observable change in terms of the electronic structure (the relative ARPES intensities can vary slightly from one location to the other) are obtained, which supports that the ARPES results are not a spatial superposition of different compositions. Such *in-situ* spatial homogeneity of the ARPES spectra

is a good indicator of a homogeneous sample. Otherwise one would have to have a "uniform" spatial inhomogeneity, i.e. a phase/stoichiometric inhomogeneity within the spot size that is uniform across the whole sample.

- **Incident photon energy dependence studies and k_z dispersion measurements**

  Supplementary Figure 3a and b show the incident photon energy dependent ARPES dispersion measurements on the $\delta = 0.40$ and $\delta = 0.525$ samples over a wide range of photon energy values. These systematic ARPES measurements show that throughout a wide range of incident photon energies from 14 eV to 70 eV, the surfaces of the samples are always dominated by the observed surface resonance band along the outer boundary of the bulk projection.

- **Demonstration of spin degeneracy at the $\bar{\Gamma}$ point**

  Here we show additional spin-resolved measurements using SR-EDC mode, which measures the net spin polarization of the electronic states as a function of binding energy at a fixed momentum value. Supplementary Figure 5 shows the SR measurements on a $\delta = 0.4$ sample using SR-EDC mode. At large momenta away from the $\bar{\Gamma}$ point (such as $k = -0.09$ Å^{-1} and $k = 0.10$ Å^{-1}), a helical spin configuration is observed. In going to smaller momenta close to the $\bar{\Gamma}$ point (such as $k = -0.03$ Å^{-1} and $k = 0.04$ Å^{-1}), the net spin polarization is found to be significantly reduced. These results are consistent with the measurements using SR-MDC mode as shown in the maintext Figs. 2 and 3. Finally at the $\bar{\Gamma}$ point at $k = 0$, no observable net spin polarization is found, which directly demonstrates the spin degeneracy of the states at the $\bar{\Gamma}$ point, consistent with the Kramers' degeneracy theorem. These systematic SR-ARPES measurements at different incident photon energies (Supplementary Figure. 4 or with different (SR-MDC or SR-EDC) measurement modes (Supplementary Figure. 5) rules out potential systematic errors in our SR measurements.

- **Model theoretical calculation results**

  We model the semi-infinite system based on the 4×4 $k \cdot p$ model [1] and utilize the Green's function method to obtain the spectral weight as well as the spin polarization near the surface region of the system as a function of bulk band-gap value in the model (see details in Supplementary Methods). Indeed, our calculation shows that, in

the conventional semiconductor regime prior to the topological transition, the spectral weight near the surface (left two panels of Supplementary Figure 6a) is dominated by a single quasi-2D band along the edge of the bulk band continuum, consistent with our ARPES results. Moreover, the calculated spin polarization in the conventional semiconductor region (left two panels of Supplementary Figure 6b) is found to be locked with momentum, also consistent with our spin-resolved measurements. Furthermore, both the calculated spectral weight and the spin polarization is found to become increasingly stronger and predominate upon approaching the TCP from the conventional semiconductor regime. A reasonable qualitative agreement between our experimental results and the $k \cdot p$ model calculation is evident as seen in Supplementary Figure 6.

- **A phenomenological picture for the preformed surface states**

  In order to better understand the spin texture of the quasi-2D states, we propose a phenomenological picture consistent with the basic topological physics for our observation: As shown in Supplementary Figures 7,8, the quasi-2D states can be viewed as a Rashba-like state, whose inner band is not observable because it is severely damped due to its strong overlap with the bulk bands in $E - k$ space (see Supplementary Figures 6,7 for a schematic). As the system is tuned approaching the TCP from the trivial side, the inner band completely loses its surface character, whereas the outer band is systematically enhanced in terms of its surface spectral weight and spin polarization, and evolve into the topological surface states (as clearly observed in our data). We emphasize that we use the term "Rashba-like" for the observed perform surface states because there are two singly degenerate bands as in a real Rashba 2DEG. However, the Rashba surface states are due to a combined effect of atomic spin-orbit coupling and the electrical field perpendicular to the surface, and follows the Rashba Hamiltonian, whereas it is not fully applicable for the observed preformed surface states. This issue needs further theoretical studies to illuminate the microscopic origin of the preformed surface states in theory.

- **Observation of preformed surface states in another topological phase transition system $(\mathbf{Bi}_{1-\delta}\mathbf{In}_{\delta})_2\mathbf{Se}_3$.**

  Here we present ARPES and spin-resolved ARPES data, which show our observation of preformed surface states in another topological phase transition system

$(Bi_{1-\delta}In_\delta)_2Se_3$. Supplementary Figure 9a shows the ARPES measured dispersion maps of the $(Bi_{1-\delta}In_\delta)_2Se_3$ for different δ compositional values. For $\delta = 0$, which corresponds to pure Bi_2Se_3, the system is clearly found to be a TI. For $\delta = 0.2$, a full bulk band-gap as large as 0.4 eV with no surface states within the bulk gap is observed, which demonstrates the conventional semiconductor (topologically trivial) nature of the $\delta = 0.2$ sample. Therefore, the system goes through a topological phase transition as the composition is changed from $\delta = 0$ to $\delta = 0.2$. Experimentally, the critical point of the topological phase transition in $(Bi_{1-\delta}In_\delta)_2Se_3$ is found to be around $\delta_c \sim 0.04$. As seen from Supplementary Figure 9b ($\delta = 0.06$), a small band-gap is found. Supplementary Figure 9c shows the spin-resolved ARPES data near the Fermi level of the $\delta = 0.06$ sample. Clear spin polarization is observed. Furthermore, the spin polarization is found to reverse as one goes from one side of the Fermi surface (cut1) to the other (cut2). These data show the observation of preformed surface states in the $(Bi_{1-\delta}In_\delta)_2Se_3$ system.

Supplementary Methods

- **EDS measurements**

In an EDS experiment, a high-energy electron beam is focused onto the surface of the sample being studied. At rest, an atom within the sample contains ground state (or unexcited) electrons in discrete energy levels or electron shells bound to the nucleus. The incident electron beam excites an electron in an inner shell (K, L, or M), ejecting it from the shell while creating a core-hole where the electron was. An electron from an outer, higher-energy shell then fills the hole, and the difference in energy between the higher-energy shell and the lower energy shell may be released in the form of an x-ray with a unique energy level characterizing a certain element. The intensity and energy of the x-rays emitted from a sample can be measured by an energy-dispersive spectrometer.

For our EDS instrument, the incident electron beam is at energy of 15 keV. The emitted x-ray photons are detected by an EDS crystal detector. Our EDS detector contains a silicon crystal that absorbs the energy of incoming x-rays by ionization, yielding free electrons in the crystal that become conductive and produce an electrical charge bias. The x-ray absorption thus converts the energy of individual x-rays into electrical voltages of proportional size; the electrical pulses correspond to the characteristic x-rays of the element. The typical energy resolution of our EDS crystal detector is about 100 eV (note that the typical core-level emitted x-ray energy is much higher, on the order of 1000eV to 10000 eV). The spatial resolution is defined by the mean free path of the incident electron beam, which is typically around 100 nm^2. This enables us to resolve the relative chemical composition in real space with a spatial resolution of 100 nm^2.

- **Model theoretical calculations**

We have created a Green's function implementation of the experimentally-based $k \cdot p$ model in Refs. [1, 2] to simulate the bulk and surface dispersions of $BiTl(S_{1-\delta}Se_\delta)_2$. Any solid with a surface can be described as a semi-infinite chain of principal layers with nearest-neighbor interactions [3]. In case of $BiTl(S_{1-\delta}Se_\delta)_2$ each unit cell represents one principal layer and the effective Hamiltonian for the unit cell is given

by

$$H_p = \begin{pmatrix} k^2/m_1 & d + k^2/m_2 & ivk_x - vk_y & 0 \\ d + k^2/m_2 & k^2/m_1 & 0 & -ivk_x + vk_y \\ -ivk_x - vk_y & 0 & k^2/m_1 & d + k^2/m_2 \\ 0 & ivk_x + vk_y & d + k^2/m_2 & k^2/m_1 \end{pmatrix}, \qquad \text{(S1)}$$

where v is the Fermi velocity, m_1 and m_2 are the orbital masses and the parameter d is introduced to generate a gap [1]. The above Hamiltonian is constructed considering two equivalent Se/S atoms (in one rhombohedral unit cell) where each Se/S atom has two p_z orbitals, one with up spin and the other with down spin. The hopping between two adjacent unit cells is realized by

$$T = \begin{pmatrix} 0 & 0 & 0 & 0 \\ t_z & 0 & 0 & 0 \\ 0 & 0 & 0 & 0 \\ 0 & 0 & t_z & 0 \end{pmatrix}, \qquad \text{(S2)}$$

where t_z is the nearest neighbor hopping parameter. To illustrate the single-Dirac-cone topological surface states, the chosen parameters are :
$m_1 = 0.125$ eV^{-1}-Å^2, $m_2 = -0.04$ eV^{-1}-Å^2, $d = -0.22$ eV, $v = 2.5$ eV-Å. The value of t_z varies with doping and we use $t_z = 0.2$ eV for the trivial side and $t_z = 0.35$ eV for the topological insulator. Now, the Hamiltonian for the semi-infinite chain can be written in a block-tridiagonal form,

$$\bar{H} = \begin{pmatrix} H_1 & T_1 & & 0 \\ T_1^\dagger & H_2 & T_2 & \\ & T_2^\dagger & H_3 & \\ & & & . \\ 0 & & & . \end{pmatrix} \qquad \text{(S3)}$$

with the surface site at 1. For the system under study, $H_1 = H_2 = H_3 = H_p$ and $T_1 = T_2 = T$. H_p and T are given in Eq. S1 and S2, respectively. A renormalization approach is used to evaluate the Green's function (GF) of this chain [4, 5]. In this approach alternate sites on the chain are eliminated and the equations for the GF

are used to define a new effective Hamiltonian with renormalized diagonal elements for the remaining sites and renormalized interactions between the remaining adjacent sites. This decimation process is repeated iteratively until the effective interaction between remaining adjacent sites is as small as one wishes. Thus, the renormalized Hamiltonian for the surface site is

$$\tilde{H}_s = H_p - T(\omega - H_p)^{-1}T^{\dagger} \tag{S4}$$

and for the bulk site is

$$\tilde{H}_b = H_p - T(\omega - H_p)^{-1}T^{\dagger} - T^{\dagger}(\omega - H_p)^{-1}T. \tag{S5}$$

The renormalized coupling between the layers is

$$\tilde{T} = T(\omega - H_p)^{-1}T. \tag{S6}$$

The Green's functions for the surface and bulk are obtained by inverting $\omega - \tilde{H}_s$ and $\omega - \tilde{H}_b$, respectively.

Now we also want to study the effect of external potential on the surface electron kinetics. When an extra potential is added at the sample surface, the surface layer and a few layers near the surface are effected, since the potential decreases rapidly as we move away from the surface towards the bulk. This effect is simulated by adding more layers on the original surface, where each layer has an appropriate potential. After adding one layer the new Hamiltonian is given by

$$\bar{H}' = \begin{pmatrix} H_0 + V_0 & T_0 & & 0 \\ T_0^{\dagger} & H_1 & T_1 & \\ & T_1^{\dagger} & H_2 & \\ & & & \cdot \\ 0 & & & \cdot \end{pmatrix}, \tag{S7}$$

where V_0 is the potential on the additional layer and $H_0 = H_p$.

The Green's function G' for the new system is calculated by using the GF for the old system. The site-diagonal GF for the new surface layer at site 0 is given by

$$G'_{00} = (\omega - (H_0 + V_0) - TG_{11}T^{\dagger})^{-1} \tag{S8}$$

and the site-diagonal GF at site 1 becomes

$$G'_{11} = (G_{11}^{-1} - T^{\dagger}(\omega - (H_0 + V_0))^{-1}T)^{-1}. \tag{S9}$$

Using this method, extra layers can be added iteratively to the chain.

[1] Fu, L. & Berg, E. Odd-Parity Topological Superconductors: Theory and Application to $Cu_xBi_2Se_3$. *Phys. Rev. Lett.* **105**, 097001 (2010).

[2] Fu, L. Hexagonal Warping Effects in the Surface States of the Topological Insulator Bi_2Te_3. *Phys. Rev. Lett.* **103**, 266801 (2009).

[3] Lee, D. H. & Joannopoulos, J. D. Simple scheme for surface-band calculations. I *Phys. Rev. B* **23**, 4988-4996 (1981).

[4] Bryant, G. W. Surface states of ternary semiconductor alloys: Effect of alloy fluctuations in one-dimensional models with realistic atoms. *Phys. Rev. B* **31**, 5166-5177 (1985).

[5] Lopez Sancho, M. P., Lopez Sancho, J. M., Sancho, J. M. L., & Rubio, J. Highly convergent schemes for the calculation of bulk and surface Green functions. *J. Phys. F: Met. Phys.* **15**, 851-858 (1985).
